# Greetings from belowground: two new species of truffles in the genus *Pachyphlodes* (Pezizaceae, Pezizales) from México

**DOI:** 10.3897/mycokeys.82.67685

**Published:** 2021-08-17

**Authors:** Carolina Piña Páez, Rosanne A. Healy, Gonzalo Guevara, Roberto Garibay Orijel, Michael A. Castellano, Efrén Cázares, James M. Trappe

**Affiliations:** 1 Instituto de Biología, Universidad Nacional Autónoma de México, Tercer Circuito s/n, Ciudad Universitaria Delegación Coyoacán, C.P. 04510, Ciudad de México, México; 2 Department of Botany and Plant Pathology, Oregon State University, Corvallis, 97331, Oregon, USA; 3 Department of Plant Pathology, University of Florida. 2550 Hull Rd, Gainesville, 32607, Florida, USA; 4 Instituto Tecnológico de Ciudad Victoria. Av. Portes Gil 1301 Poniente, C.P. 87010 Ciudad Victoria, Tamaulipas, México; 5 USDA Forest Service, Northern Research Station, 3200 Jefferson Way, Corvallis, 97331, Oregon, USA; 6 Department of Forest Ecosystems and Society, Oregon State University, Corvallis, 97331, Oregon, USA

**Keywords:** Ascomycota, hypogeous, new taxa, sequestrate fungi, systematics, truffles

## Abstract

*Pachyphlodes* is a lineage of ectomycorrhizal, hypogeous, sequestrate ascomycete fungi native to temperate and subtropical forests in the Northern Hemisphere. *Pachyphlodes* species form ectomycorrhizae mainly with Fagales hosts. Here we describe two new species of *Pachyphlodes*, *P.brunnea*, and *P.coalescens*, based on morphological and phylogenetic analysis. *Pachyphlodesbrunnea* is distributed in the states of Tamaulipas and Nuevo León in northern México, occurring with *Quercus* and *Juglans* species. It is characterized by its dark brown peridium, white gleba, and spores with capitate columns. *Pachyphlodescoalescens* is distributed in the states of Michoacán and Tlaxcala in central and southwestern México co-occurring with *Quercus* and is distinguished by its reddish-brown peridium, light yellow gleba, and spore ornamentation. Both species, along with *P.marronina*, constitute the Marronina clade. This clade contains North American species characterized by a brown peridium and spores ornamented with capitate spines to coalesced spine tips that form a partial perispore.

## Introduction

*Pachyphlodes* Zobel, 1854 (Pezizaceae, Pezizales) is characterized by truffle-like ascomata with a thick peridium of large isodiametric cells and globose spores ornamented with spines or columns. The spores are either naked or covered with a perispore (Tulasne and Tulasne 1844; Healy et al. 2018). There are currently 16 recognized *Pachyphlodes* species and two varieties in the genus (Kirk 2016; Ting et al. 2019). *Pachyphlodes* was known as *Pachyphloeus*, but this name was declared illegitimate (Healy et al. 2018), so its species were transferred to the oldest legitimate name *Pachyphlodes* (Doweld 2013a, b). *Pachyphlodes* species are distributed across the Northern Hemisphere; in North America and Europe; they form ectomycorrhizae with hosts in the Betulaceae, Fagaceae, and Juglandaceae in temperate and subtropical regions (Smith et al. 2007; Lindner and Banik 2009; Stefani et al. 2009; Tedersoo et al. 2009, 2010; Bonito et al. 2011; García-Guzmán et al. 2017). With the use of molecular techniques, the number of *Pachyphlodes* species has nearly doubled from eight species and two varieties in 2000 to 16 species and two varieties in 2020. Four species have been described from México; Cázares et al. (1992) reported *P.citrina* (Berk. and Broome) Doweld (unverified by molecular methods) from Nuevo León and *P.virescens* (Gilkey) Doweld (unverified by molecular methods) from Nuevo León and Tamaulipas; Healy et al. (2009) reported Pachyphlodescf.carnea from Nuevo León, and described a new species *P.marronina* Healy, Bonito & Guevara from Nuevo León, Tamaulipas and Tlaxcala. Healy et al. (2009) remarked on morphological differences between the *P.marronina* collections from the upper Midwestern USA and the *P.marronina* collections from México and proposed they may be part of a species complex in need of further analysis. With the aim to solve this species complex, here we report new collections along with results from further analyses that support the description of the Mexican collections as two new species of *Pachyphlodes* in the Marronina clade.

## Materials and methods

### Morphological observations

Ascomata of *P.brunnea* were collected from the state of Tamaulipas, while *P.coalescens* collections were found across the states of Michoacán and Tlaxcala. All the specimens are deposited in the following herbaria: Oregon State University (**OSC**), Instituto Tecnológico de Ciudad Victoria (**ITCV**) and Herbario Nacional de México (**MEXU**). Macroscopic characters were described from fresh specimens under natural light, and colors of fresh ascomata are described in general terms by the authors. Microscopic characters were described from razor-blade sections of fresh specimens mounted in 5% KOH and Melzer’s reagent. Fifty measurements were taken per structure; measurements of structures are length by width (this is the order of appearance in the descriptions). For scanning electron microscopy (SEM), ascospores were scraped from the dried gleba onto double-sided tape, which was mounted directly on an SEM stub, coated with platinum-palladium, and examined and photographed with a HITACHI TM 3000 scanning electron microscope, or they were prepared and imaged as outlined in Healy et al. (2018).

### DNA sequencing and phylogenetic analyses

A tissue sample from collection MEXU 26842 was sent to the Canadian Center of Barcoding (CCDB) for extraction, amplification, and sequencing of the Internal Transcribed Spacer (ITS). DNA was extracted from JT32454, JT32623, and ITCV-GGG-896 at the University of Minnesota with a modified CTAB method (Healy et al. 2009). The ITS1-5.8s-ITS2 (ITS) region was amplified with ITS1 and ITS4 (White et al. 1990) and ITS1f (Gardes and Bruns 1993). DNA sequences were deposited in GenBank (Table 1). Sequences were edited in Geneious 7.1 (Kearse et al. 2012) or Sequencher 4.0 (Gene Codes, Ann Arbor, MI). As done in Piña Páez et al. (2018), the distribution of species was complemented with soil DNA data from central and south México through a BLASTn search against the Mexican Soil Fungi Database in Geneious 10.1. This database includes ITS2 sequences of soil fungi from México and has been partially published in Argüelles-Moyao and Garibay-Orijel (2018).

**Table 1.** Accession and voucher numbers of sequences included in the phylogenetic analysis. Herbarium collection with * indicates holotypes and ** indicates paratypes.

Species	Herbarium	Country	GenBank
*Amylascus*	OSC:H5626	Australia	JX414224, KJ720812
*Amylascus*	MEL2364119A	Australia	KT318375
*Pachyphlodesannagardnerae*	ISC:RH46*	USA: IA	JN102472
	ISC:RHAM14	USA: IA	JN102375
*Pachyphlodesaustro-oregonensis*	SOC775*	USA: OR	JX414191
*Pachyphlodesbrunnea*	ITCV896*	Mexico	HQ324990
	JG3757	Mexico	EU427551
	OSC:JT32623	Mexico	MT461399
	DUKE	Mexico	JN102443
*Pachyphlodescarnea*	OSC43593	USA: CA	JX414189
	FLAS-F-63788	USA: CA	MT461396
*Pachyphlodescinnabarina*	HMAS-96735*	China	MK192830
	BJTC-FAN946	China	MK192831
	BJTC-FAN1157	China	MK192829
*Pachyphlodescitrina*	FLAS:JBP-2011-09-10	France	KJ720747
	FLAS-F-59182	England	JN102468
	OSC:JRWL 2197	Italy	EU543196
*Pachyphlodescoalescens*	MEXU-26842*	Mexico	KJ595000
	TXLM:JT32454	Mexico	EU543209
*Pachyphlodesconglomerata*	FLAS-F-66164	Spain	KJ720788
	MA-29354	Spain	JN102487
*Pachyphlodesdepressa*	BJTC:FAN302*	China	KP027405
	BJTC:FAN324	China	KP027406
*Pachyphlodesligerica*	FLAS-F-62613	France	MT461402
*Pachyphlodesmarronina*	MIN-925598	USA: IA	KJ720786
	MIN-925612	USA: IA	JN102364
	HUH-258432*	USA: IA	EU427549
*Pachyphlodesmelanoxantha*	FLAS-F-61135	England	JX414217
	FLAS-F-66172	France	KJ720792
	FLAS-F-66167	Spain	KJ720793
*Pachyphlodesnemoralis*	FLAS-F-61964	France	MT461400
	FLAS-F-66166	Spain	MF462328
	FLAS-F-59181*	England	JN102469
	S-F-133989	Sweden	JX414218
*Pachyphlodesoleifera*	FLAS-F-64137	Spain	KJ720787
	MA-82461*	Spain	JQ996421
*Pachyphlodespfisteri*	FLAS-F-59179*	USA: ME	JN102474
*Pachyphlodesthysellii*	OSC 80959**	USA: WA	EU543197
	FLAS-F-66243	USA: MN	JN102479
*Pachyphlodesvirescens*	FLAS-F-60565	USA: CA	MT461401
	OSC JT13043	USA: CA	JX414219
*Pachyphlodeswulushanensis*	BJTC-FAN923*	China	MK192827

Phylogenetic analyses of ITS rDNA have been implemented to describe and resolve species delimitation in *Pachyphlodes* (Healy et al. 2015; Li et al. 2019; Liu et al. 2020). Phylogenetic analyses utilizing the 28S rDNA, ß-Tubulin, and RPB2 markers showed that *Pachyphlodes* is a member of the *Pezizaceae*, that *Plicariella* (Sacc.) Rehm (as *Scabropezia* Dissing and Pfister) is sister to *Pachyphlodes*, and that the sister lineage to *Pachyphlodes* and *Plicariella* is *Amylascus* Trappe (Hansen et al. 2005). Healy et al. (2018) showed that *Plicariella* is within or sister to the Melanoxanthus clade of *Pachyphlodes*. Our phylogenetic analysis consisted of 42 sequences from 16 described species, including nine sequences from type specimens of *Pachyphlodes* and from *Amylascus* Trappe. *Amylascus* was selected as an outgroup based on previous phylogenetic analyses. DNA sequences were aligned with MAFFT v 6.822 (Katoh and Toh 2010) and manually improved in SE-AL v2.0a11 (Rambaut 2007) for a final alignment with 754 positions. Phylogenetic inferences were estimated with maximum likelihood in RAxML 7.2.8 (Stamatakis 2006) with a GTR + G model of nucleotide substitution. For Bayesian posterior probability, priors were selected with jModeltest 2.1.4 (Darriba et al. 2012), under the Aikake information criterion, and posterior probability was estimated in MrBayes 3.1.2 (Huelsenbeck and Ronquist 2001) with 20,000,000 generations with trees sampled every 1000 generations. The first 25% of samples were discarded as burn-in, and stationarity was checked in Tracer (Rambaut and Drummond 2007). RAxML and MrBayes were both runs on the Cipres Portal (Miller et al. 2010). Trees were visualized and optimized in FigTree 1.4.4 (http://tree.bio.ed.ac.uk/software/figtree/), and font and color were added in Adobe Illustrator vCS4 (Adobe Systems, Inc., San Jose, CA). Alignment is available in OSF (Open Space Framework, to be uploaded prior to journal submission).

## Results

**Figure 1.**
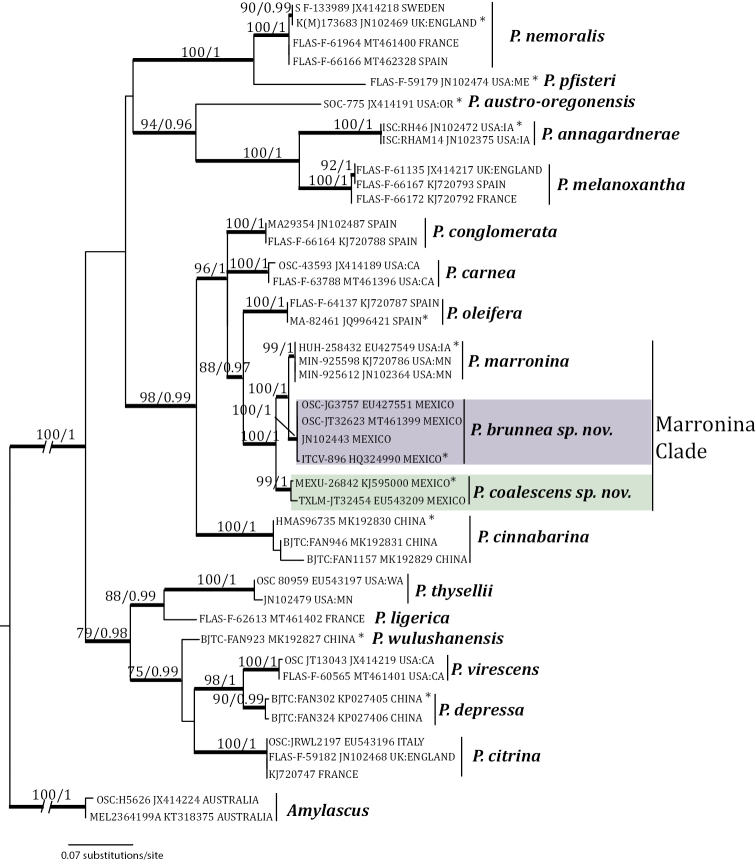
The most likely tree generated from RAxML analysis of the ITS sequences of 18 *Pachyphlodes* species, rooted with *Amylascus*. Thickened branches denote >70% bootstrap support (left of slash) and >0.95 posterior probability (right of slash) from Bayesian analysis. New species are in shaded boxes, and Marronina clade demarcated. Terminals contain GenBank accession number, herbarium number, and country/state of collection. Asterisks denote sequences from holotypes.

The nucleotide substitution model selected by jModeltest was TPM1uf+I+G. The final optimization likelihood was –lnL 4774.259669, and the most likely tree is shown in Fig. 1. Both the Maximum Likelihood and Bayesian analyses (Fig. 1) show that *P.brunnea* forms a new strongly supported clade (100/1), which includes sequences from voucher collections and ectomycorrhizae. This clade is placed as a sister taxon of *P.marronina*, which also forms a strongly supported clade (99/1). The sister taxon (100/1) of these two species is *P.coalescens*, which is also a new strongly supported clade (99/1).

### Taxonomy

#### Pachyphlodes brunnea

Taxon classificationFungiPezizalesPezizaceae

Guevara, Piña Páez & Healy sp. nov.

C5613BB5-0DBC-5057-8AD8-AD05D8587F1E

835665

ITS barcode GenBank: HQ324990 (Holotype), EU427551, MT461399, JN102443

Fig. 2a–d

##### Type.

México, Tamaulipas, Ciudad Victoria, Torre de Microondas “Las Mulas”, 23°37'00"N, 99°14'31"W, alt. 1549 m, under *Quercuspolymorpha* Schlecht. & Cham., *Quercus* sp. and *Juglans* sp., hypogeous, solitary or in groups of 2, 11 November 2006, col. G. Guevara (holotype: ITCV 896).

##### Diagnosis.

*Pachyphlodesbrunnea* is be recognized by the dark brown ascomata and two-layered. Thick (474–570 µm) peridium, white gleba when immature, spores ornamented with capitate columns growing under *Quercus* and with an odor similar to raw potatoes.

##### Etymology.

Latin, brunnea in reference to the brown peridium.

##### Description.

**Figure 2.**
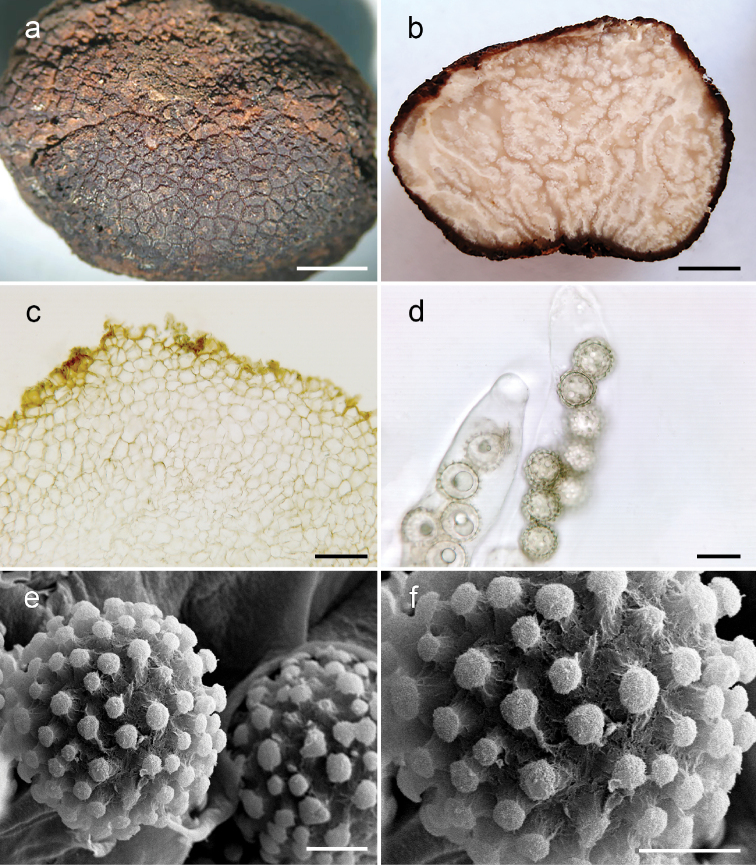
*Pachyphlodesbrunnea* (Holotype: ITCV 896) **a** ascoma dried **b** gleba in cross-section **c** peridium in cross-section, showing a wart composed of isodiametric cells **d** light microscopy of asci and spores **e, f**SEM microscopy of spores in surface view. Scale bars: 3 mm (**a, b**), 20 µm (**c, d**), 5 µm (**e, f**).

***Ascomata*** subglobose to ovoid, 15–17 × 10–15 mm, surface dry, with an irregular basal depression, surface dark brown when fresh (Fig. 2a), with geometric, angular, or pyramidal warts 1 mm wide, with flattened, elevated, or rounded top. Gleba solid (Fig. 2b), marbled with white sterile veins separating brownish, fertile tissue, overall brownish when dried. Odor of corn starch-like or of raw potatoes.

***Peridium*** of two layers. Outer peridium 125–570 µm thick, of textura angularis, with warts up to 300–500 (–800) μm high, outermost cells up to 42 μm broad, some ventricose or irregular, radial arrangement in some areas, walls 2–3 (–5) μm thick, reddish-brown to orange-brown in 5% KOH, innermost cells up to 10 μm broad, walls 1–2 μm thick, hyaline in 3% KOH. Inner peridium 120–500 (–700) μm thick, composed of hyaline, septate, interwoven hyphae (textura intricata), 5–12 µm broad, thin-walled 1–2 μm thick. ***Asci*** 8-spored, clavate, subclavate, subfusoid or irregular, 120–238 × 30–45 µm including pedicel, hyaline in 5% KOH, walls 1 µm thick, asci are scattered. ***Paraphyses*** not detected. ***Ascospores*** irregularly biseriate to uniseriate, hyaline in 5% KOH, globose, including ornamentation 18–22 µm broad, mean = 20 µm; excluding ornamentation 12–18 (–20) µm broad, mean = 15 µm. Ornamentation averaging 1.5 (–2.0) µm high, capitate columns, consisting of columns with a boarder, rounded tip.

##### Distribution and ecology.

Known only from northeastern México (Tamaulipas, Nuevo Leon). Ascomata hypogeous always associated with *Quercuspolymorpha*, and DNA (JN102443) of this species were recovered from sampled roots of oak (JN102443) from Chipinque National Park in Nuevo León. No DNA sequences of this species were found in soil in central or southern México.

##### Specimens examined.

México, Tamaulipas, Ciudad Victoria, Torre de Microondas “Las Mulas”, 23°37'00"N, 99°14'31"W, alt. 1549 m, under *Quercuspolymorpha*, *Quercus* sp. and *Juglans* sp., hypogeous, solitary or in pairs, November 11, 2006, col. G. Guevara (ITCV 891; No ITS); Carretera Victoria, El Madrono, 23°36'3"N, 99°13'8"W, alt. 1460 m, under *Quercuscanbyi* Trel., *Q.polymorpha*, and *Q.laeta* Liebm., hypogeous, August 1, 2008, col. G. Bonito (JT32623; GenBank MT461399). Nuevo León, Municipio de Santiago, El Cercado September 14, 1983, col J. García (UNL 3757; GenBank EU427551).

##### Taxonomic comments.

The ITS sequences of *Pachyphlodesbrunnea* are similar to those of *P.marronina* (97.79% of identity and 12 nucleotide differences in ITS region), which is why it was originally described as *P.marronina*. However, the peridium color and geographic location of these two species differ considerably. Spore ornamentation also separates them. The fresh peridium of *P.marronina* is red with indistinct warts, while that of *P.brunnea* is dark brown with distinct angular warts. The angular to pyramidal warts in the peridium of *P.brunnea* aretaller (300–800 µm) than the lower, indistinct warts on *P.marronina* (160–270 µm). The spines in *P.marronina* are taller (1.5–3.0 µm) than *P.brunnea* (1.5–2.0 µm), conferring a different aspect to the spores overall (Fig. 2e, f). *Pachyphlodesbrunnea* superficially resembles *P.melanoxantha* (Tul. & C. Tul. ex Berk.) Doweld and *P.annagardnerae* R.A. Healy & M.E. Sm., but the latter two are black to the unaided eye, purple under transmitted light, have acute tipped spiny spores, and *P.melanoxantha* is said to have a nauseous odor (Berkeley 1844). In contrast, *P.brunnea* is dark brown to the unaided eye, yellowish-brown under transmitted light, and has a pure white gleba with capitate spore spines and a pleasant odor. *Pachyphlodesannagardnerae* has no perceptible odor.

#### Pachyphlodes coalescens

Taxon classificationFungiPezizalesPezizaceae

Piña Páez, R.A. Healy & Cázares sp. nov.

CDBAA734-339F-5B1D-ACDE-AF77088418CD

835666

GenBank KJ720784, KJ595000 (Holotype).

Fig. 3 a–e

##### Type.

México, Michoacán, road Morelia-Atécuaro, Morelia, 19°36'0"N, 101°10'58.8"W, alt. 2280 m, under *Quercusdeserticola* Trel., hypogeous, solitary, 30 September 2012, col. R. Garibay-Orijel (holotype: MEXU 26842).

##### Diagnosis.

*Pachyphlodescoalescens* can be recognized by the brown ascomata and two-layered, thick (600–700 µm) peridium, and a gleba marbled with light yellow, meandering, sterile veins alternating with dark brown fertile veins, spores ornamented with truncated spines, that have material deposited at the tips, which accumulates and coalesces with neighboring tip material to form a broad, meandering, roughened, reticulum that hides the underlying spines, growing under *Quercus*.

##### Etymology.

Named for the process that produces the spore ornamentation: material deposited on the spine tips coalesces to form a meandering reticulum, from Latin coalecere, to grow together.

##### Description.

**Figure 3.**
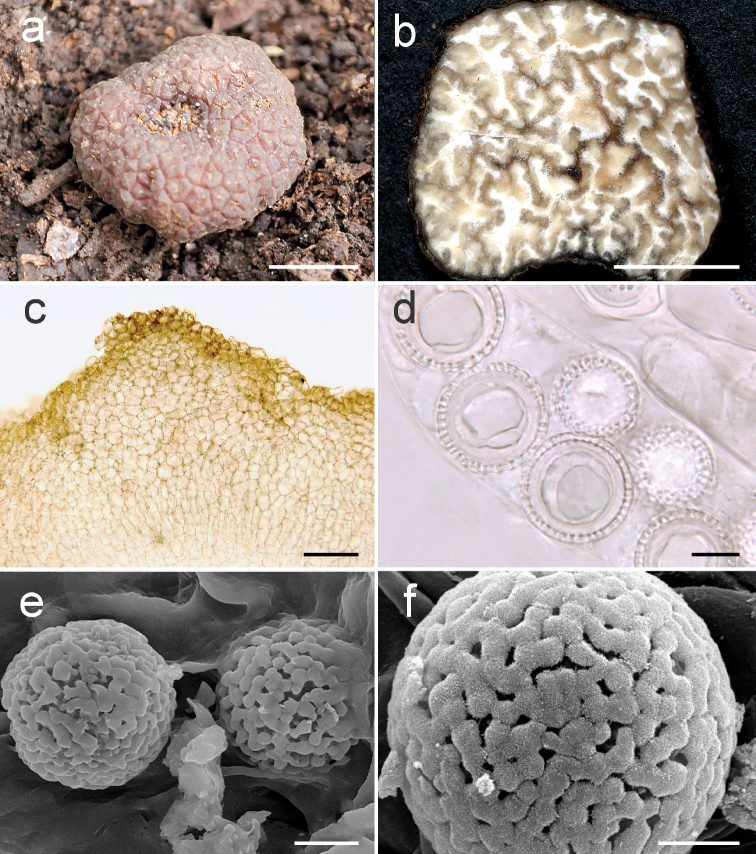
*Pachyphlodescoalescens* (Holotype: MEXU 26842) **a** ascoma fresh **b** gleba in cross-section **c** peridium in cross-section, showing a wart composed of isodiametric cells **d** light microscopy of asci and spores **e, f**SEM microscopy of spores in surface view. Scale bars: 5 mm (**a, b**), 100 µm (**c**), 10 µm (**d, e**), 5 µm (**f**).

***Ascomata*** irregularly subglobose, slightly compressed, 12 × 14 mm, surface with flat, polygonal warts with 4–6 sides, each wart about 2.5–3.0 mm broad, orange-brown when fresh (Fig. 3a), dark reddish-brown when dried, areole 6 × 4 mm where internal sterile veins emerge. Gleba light yellow with translucent yellowish sterile veins when fresh becoming cream with light brown veins when dried (Fig. 3b).

***Peridium*** of two layers. Outer peridium 440–500 μm thick, composed of textura angularis, with warts up to 220 μm high, outermost cells up to 30 μm broad, walls 1 μm broad, orange-brown in 5% KOH, interior cells up to 22 μm broad with notably thinner cell walls <0.5 µm, hyaline (Fig. 3c). Inner peridium about 175–190 μm thick, composed of hyaline, septate, interwoven hyphae 4.5–6.5 µm broad, thin-walled <0.5 μm. ***Paraphyses*** filiform, septate, with swollen tips, 200–210 × 8.75 μm, 10–14 μm broad at the apex, pale green with granular contents, thin-walled <0.5 μm. ***Asci*** 8-spored, irregularly distributed in fertile brown veins among interwoven hyphae, pyriform to cylindrical with a short pedicel, 180–195 μm long including pedicel, 40–50 μm wide, pedicel 22–26 × 10–12 μm, widening at the base, hyaline in 5% KOH, walls <0.5 µm (Fig. 3d). Spores irregularly biseriate to uniseriate. No reaction of asci in Melzer’s reagent. ***Ascospores*** (Fig. 3e, f) globose, hyaline to light yellow, size range including ornaments 20–23 μm, averaging 21.20 μm, spores excluding ornaments 16–18 μm, averaging 17.70 μm. Ornamentation averaging 1.80 μm high, of short capitate spines that accumulate material at the tips that coalesces to produce a nearly solid covering over the spore by maturity.

##### Distribution and ecology.

Ascomata hypogeous, known from Michoacán and Tlaxcala co-occurring with *Quercusdeserticola* Trel, *Quercusrugosa* Née, and *Q.crassifolia* Humb. & Bonpl. DNA sequences have also been found in *Quercus* dry forests or xerophilous pine-oak forests in Libres in Puebla, Tequila volcano in Jalisco, and Cerro del Águila in Michoacán, all in central-southwestern México.

##### Specimens examined.

**México**, Tlaxcala, 1 km east of San Francisco Temezontla, Municipio Panotla, alt. 2600 m, under *Quercusrugosa* Née, and *Q.crassifolia* Humb. & Bonpl., September 20, 2007, col. E. Cázares (JT32454; GenBank EU543209).

##### Taxonomic comments.

*Pachyphlodescoalescens* has a texture and peridial structure of the peridium similar to the other two species of the Marronina clade (*P.brunnea* and *P.marronina*) clade, but they vary in other macroscopic or microscopic characteristics. Ascomata of *Pachyphlodesbrunnea* are dark brown to brownish black, whereas *P.coalescens* ascomata are orange-brown. In addition, they differ in spore size (*P.brunnea* 18–22 μm vs. *P.coalescens* 20–23 μm), and the spore ornamentation of *P.brunnea* is of discreet, capitate columns, whereas in *P.coalescens*, it is of spines with additional material that is so thickly deposited at the apices as to form a broad, meandering perispore that nearly covers the spore surface. *Pachyphlodescoalescens* are similar to *P.marronina*, but the latter has smaller spores (19–22 μm) ornamented with coarse, mostly discreet, truncate to capitate spines, whereas *P.coalescens* has short spines fully connected at the tips via the material deposited at the apex of each spine (see above). The spore ornamentation of *P.coalescens* is similar to that of *P.nemoralis* Hobart, Bóna & A. Paz and *P.pfisteri* Tocci, M.E. Sm. & Healy, which otherwise differ strongly in color, peridium structure, and phylogenetic placement.

## Discussion

The *Pachyphlodesmarronina* original description included collections from Iowa, U.S.A., Nuevo León and Tlaxcala, México. Cryptic diversity within this species was addressed by Healy et al. (2009) concerning molecular differences between the *P.marronina* collection from the U.S. and the Mexican collections. We now have additional molecular, geographical, and morphological evidence that the *P.marronina* complex includes three distinct species across North America: *P.brunnea*, *P.coalescens*, and *P.marronina*. Our evidence indicates that *P.brunnea* is associated with *Quercus* on the basis of molecular information from an ectomycorrhizal sequence (JN102443). However, no direct evidence exists that *Quercus* is the ectomycorrhizal host for the other two species. Their habitat descriptions suggest these two species associate with *Quercus*, but we need more environmental data to corroborate the association.

The three members of the Marronina clade (Fig. 1) have an ornamented peridium with flat warts, which are more conspicuous in *P.brunnea* and *P.coalescens*. The structure and composition of the peridium are also similar; all three have a two-layered peridium composed of an outermost layer of textura angularis and an inner layer of texture intricata. The biseriate to uniseriate arrangement of the spores in the asci is similar across the three species. *Pachyphlodesbrunnea* resembles *P.marronina* in spore ornamentation; both species have spores with spines columns that are joined by the accumulation of material at the apex of the spines. However, the shorter spines in *P.brunnea* confer a clumpier appearance overall. The spore ornamentation in *P.coalescens* is different from the other two Marronina clade members but is simply the result of the coalescence of spine tip material, which occurs only occasionally in *P.marronina*. The late-stage spore ornamentation of the coalescence of spore tip material is also seen in *P.nemoralis* and *P.pfisteri* (Healy et al. 2015), but these species otherwise differ in color, peridium structure, and phylogenetic placement (Fig. 1).

The sister species of the Marronina clade is *P.oleifera* (Fig. 1), a European taxon with very distinct morphological characters, peridium with coarse warts, an unusual gray blueish gleba, and finely verrucose spores (Cabero and Pérez-Pérez 2012). Another characteristic of *P.oleifera* that separates it from the rest of the known species of *Pachyphlodes* is the oily content in all the microscopic structures, particularly hymenial cells.

## Supplementary Material

XML Treatment for Pachyphlodes brunnea

XML Treatment for Pachyphlodes coalescens
